# Inhibiting UCH-L5: Rational Design of a Cyclic Ubiquitin-Based Peptide Inhibitor

**DOI:** 10.3389/fmolb.2022.866467

**Published:** 2022-05-26

**Authors:** Dharjath S. Hameed, Huib Ovaa, Gerbrand J. van der Heden van Noort, Aysegul Sapmaz

**Affiliations:** Oncode Institute and Department of Cell and Chemical Biology, Leiden University Medical Centre, Leiden, Netherlands

**Keywords:** ubiquitin, cyclic peptide, UCH-L5, peptide inhibitor, deubiquitinase, proteasome

## Abstract

The ubiquitin-proteasome system is an essential regulator of many cellular processes including controlling protein homeostasis. The degradation of proteins by the multi-subunit proteasome complex is tightly regulated through a series of checkpoints, amongst which are a set of deubiquitinating proteases (DUBs). The proteasome-associated DUBs, UCH-L5 (Ubiquitin carboxyl-terminal hydrolase isozyme L5) and USP14 (Ubiquitin-specific protease 14), and the integral-DUB in the proteasome, Rpn11, is known to regulate proteasomal degradation by deubiquitination of distinct substrates. Although selective inhibitors for USP14 and Rpn11 have been recently developed, there are no known inhibitors that selectively bind to UCH-L5. The X-ray structure of the Ubiquitin (Ub) bound to UCH-L5 shows a β-sheet hairpin in Ub that contains a crucial hydrophobic patch involved in the interaction with UCH-L5. Herein, we designed and developed both a Ub sequence-based linear- and cyclic- β-sheet hairpin peptide that was found to preferably inhibit UCH-L5. We show that these peptides have low micromolar IC_50_ values and the cyclic peptide competes with the activity-based UbVME (Ubiquitin-Vinyl-Methyl-Ester) probe for UCH-L5, binding in a concentration-dependent manner. We further establish the selectivity profile of the cyclic peptide for UCH-L5 compared to other members of the UCH-DUB family and other cysteine DUBs in cell lysate. Furthermore, the cyclic peptide infiltrated cells resulting in the accumulation of polyUb chains, and was found to be non-toxic at the concentrations used here. Taken together, our data suggest that the cyclic peptide permeates the cell membrane, inhibits UCH-L5 by possibly blocking its deubiquitinating function, and contributes to the accumulation of polyubiquitinated substrates. The implications of inhibiting UCH-L5 in the context of the 26S proteasome render it an attractive candidate for further development as a potential selective inhibitor for therapeutic purposes.

## Introduction

Ubiquitination is a crucial post-translational modification (PTM) involved in various cellular processes (Komander et al., 2009; Ciechanover, 2015), tightly regulated by a complex myriad of ubiquitinating enzymes. The Ub-conjugation cascade involves Ub-activating E1 enzymes, conjugating E2 enzymes that either transfer Ub directly to the substrate via a scaffolding function of a RING (Really Interesting New Gene) E3 ligase enzyme, or transfer the Ub cargo to a HECT (Homologous to E6-AP Carboxyl Terminus) or RBR (Ring-Between-Ring) E3-ligase to finally attach the Ub to (mostly a lysine residue in) the targeted substrate protein (Hochstrasser, 1996). The dynamic nature of this PTM is co-regulated by deubiquitinating protease enzymes called deubiquitinases (DUBs), reversing the (poly)ubiquitinated state of the substrate (Hochstrasser, 1996). Substrates can be modified with a single Ub or multiple Ubs by conjugation of Ub to specific lysine residue or N-terminus of the substrate. Additionally, Ub can be modified via its seven internal lysine residues or methionine residue to form eight homotypic polyUb chain types that can be further extended to a combination of heterotypic or branched polyUb chains (Yau and Rape, 2016; Perez Berrocal et al., 2019; French et al., 2021). One of the most prevalent forms of ubiquitination is the modification of substrates with Lys48 linked polyubiquitin chains, marking the substrate for degradation via a multi-subunit protease complex called the 26S proteasome (Chau et al., 1989; Yau and Rape, 2016). The human 26S proteasome is made up of a 20S core particle (CP) flanked by one or two 19S regulatory particles (RP). The CP has a pair of hetero-dimeric rings composed of seven α (1–7) and seven β (1–7) subunits. The peptidases that cleave amide bonds in the substrate protein reside in this complex. The RP, on the other hand, acts as a gateway and consists of 13 Rpn subunits and 6 ATPases that facilitate the transfer of the uncoiled amino acid backbone of the substrate into the CP (Bard et al., 2018). The different subunits of the 26S proteasome work in unison to facilitate effective protein degradation (Collins and Goldberg, 2017), resulting in shorter peptides or amino acids that are destined either for recycling or for immunological functions such as antigen presentation (Grant et al., 1995).

Given the highly promiscuous nature of proteasomal degradation, the 26S proteasome is tightly regulated by kinases, phosphatases, and deubiquitinases amongst others (de Poot et al., 2017; VerPlank and Goldberg, 2017). The 26S proteasomal DUBs include UCH-L5, USP14 (known as Ubp6 in yeast), and POH1/PSMD14 (known as Rpn11 in yeast) (Lee et al., 2011; de Poot et al., 2017). UCH-L5 and USP14 are part of the cysteine-protease class of DUBs dynamically associated with the proteasome complex, while Rpn11 is a metalloprotease that is an integral part of the lid complex (19S) of the 26S proteasome (Selvaraju et al., 2015; Bard et al., 2018). The consolidated structure of the 26S proteasome has shown that UCH-L5 associates with the Rpn13 subunit of the 19S lid complex of the proteasome (de Poot et al., 2017). The DEUBAD (DEUBiquitinase Adaptor) domain of Rpn13 is essential to the binding of UCH-L5 and triggers the proteasome-associated deubiquitinating activity of UCH-L5. In the case of UCH-L5 found in the nucleus, a nuclear protein called Nuclear factor related kappa-B-binding protein (INO80G/NFRKB) inhibits the deubiquitinating activity of UCH-L5 by mimicking the ubiquitin-binding using its DEUBAD domain, thus, regulating the UCH-L5 activity in cells. (Sahtoe et al., 2015).

Aberrant proteasome-mediated degradation is one of the hallmarks of cancer progression (Mani and Gelmann, 2005; Patel et al., 2018). Targeting DUBs associated with the 26S proteasome, hence, is actively being explored as an attractive therapeutic strategy. Over the years, small molecule inhibitors such as IU1 targeting USP14, bAP15 targeting both USP14 and UCH-L5 (D'Arcy et al., 2011; Nag and Finley, 2012), and recently, Capzimin and thiolutin targeting Rpn11 have been developed (Lauinger et al., 2017; Li et al., 2017). However, inhibitors selectively targeting UCH-L5 are still lacking (Shin et al., 2020). Although identifying small molecule inhibitors that target UCH-L5 is a viable option, most of these molecules are covalent electrophiles that can be promiscuous by targeting active-site cysteines of other DUBs and cysteine proteases (Kemp, 2016; Harrigan et al., 2018). Hence we deemed peptide-based inhibitors, currently unexplored as DUB inhibitors, as an interesting option since such inhibitors can be designed based on structural information, potentially providing a head-start with a higher degree of selectivity towards the target (Lee et al., 2019). With this rationale in mind, we set out to develop a peptide-based inhibitor for UCH-L5 that binds to an allosteric region rather than the active site cysteine. We first designed a beta-sheet hairpin peptide based on the Ub sequence, consisting of residues 1 to 17 that include the hydrophobic patch comprising Leu8 and Thr9, as identified from the reported structure of UCH-L5/Rpn13:Ub (Sahtoe et al., 2015; Vander Linden et al., 2015). Using a solid-phase peptide synthesis (SPPS) strategy (Huppelschoten and van der Heden van Noort, 2021) we synthesized both the end-protected linear version of the beta-sheet hairpin of Ub consisting of residues 1 to 17 as well as a cyclic version of the same. We deemed that both end-protection and cyclization of the peptide would stabilize the beta-sheet conformation and enhance proteolytic stability in cells. Both the linear and the cyclic peptide showed an inhibitory effect on UCH-L5 enzymatic activity while a mutant peptide lacking the hydrophobic patch (Leu8 and Thr9) did not inhibit at all. We observed that the cyclic peptide inhibited UCH-L5 at lower micromolar concentrations compared to other DUBs in the UCH family even though they share a similar binding mode to Ub. Furthermore, we observed that the peptide competed with the activity-based Ub-Vinylmethylester (UbVME) and Ub-propargylamide (UbPA) probe, thus establishing allosteric inhibition of UCH-L5. We further demonstrate the selectivity profile for UCH-L5 compared to other DUBs. Moreover, we show that the treatment of cells with our cyclic peptide results in the accumulation of ubiquitinated substrates without any toxicity on the cells caused by the peptide, suggesting that the cyclic peptide permeates the cell membrane and potentially inhibits the activity of UCH-L5.

## Materials and Methods

### General Methods

All commercial materials (Aldrich, Fluka, Novabiochem) were used without further purification. All solvents were reagent grade or HPLC grade. LC-MS analysis was performed on a system containing a Waters 2795 separation module (Alliance HT), Waters 2996 Photodiode Array Detector (190–750 nm), Phenomenex Kinetex XB-C18 (2.1 × 50 mm) reversed-phase column, and a Micromass LCT-TOF mass spectrometer. Samples were run at 0.80 ml/min with the use of a gradient of two mobile phases: A) aq. formic acid (0.1%), and B) formic acid in CH_3_CN (0.1%). Data processing was performed using Waters MassLynx 4.1 software. Preparative HPLC was performed on a Waters XBridge™ Prep C18 Column (30 × 150 mm, 5 μm OBD™) at a flow rate of 37.5 ml/min using aq. 0.05% TFA (Solvent A) and acetonitrile containing 0.05% TFA (Solvent B) as eluents. For purification of peptides, a gradient from 25% B to 95% B over 18 min was used. All samples containing pure peptides were pooled and lyophilized.

### Fmoc-SPPS of Peptides

SPPS was performed on a Syro II MultiSyntech Automated Peptide synthesizer using standard 9-fluorenylmethoxycarbonyl (Fmoc) based solid-phase peptide chemistry at 20 µmol scales. All amino acids were used in 5-fold excess and coupling mix containing Benzotriazole-1-yl-oxy-tris-pyrrolidino-phosphonium hexafluorophosphate (PyBOP) and *N*,*N*-Diisopropylethylamine (DIPEA) were used accordingly. Linear Ub_1-17_ peptide was synthesized on H-Rink amide Chemmatrix^®^ resin (Sigma-Aldrich), and the N-terminus of this peptide was capped with an acetyl group using acetic anhydride. After synthesis, the peptide was completely deprotected using Trifluoroacetic acid (TFA) cleavage mix for 2 h (TFA: H_2_O: Triisopropylsilane (TiPS): Phenol (92.5/2.5/2.5/2.5 v/v/v/v)). The cyclic peptide Ub_1-17_ was synthesized on TentaGel Trt R resin with a free N-terminus. Immediately after synthesis, the cyclic peptide was cleaved from the resin using Hexafluoroisopropanol (HFIP) in Dichloromethane (DCM, 20% v/v) so that the side chains remain protected while the N- and C-terminus are liberated. Cyclization was carried out using 1.2 equiv. PyBOP, 1.4 equiv. DIPEA in Dimethylformamide (DMF) at a peptide concentration of 0.5 mg/ml, overnight at room temperature. After cyclization, the peptide was completely deprotected using the same TFA cleavage mix used for the linear peptide. Following deprotection, the peptide was precipitated in the cold ether:pentane (v:v 1:1) and lyophilized. The peptides were then dissolved in aqueous Dimethylsulfoxide (DMSO, 5%) and purified twice using preparative RP-HPLC. All purified peptides **1**, **2,** and **3** were lyophilized and dissolved in DMSO before being stored at -20°C.

### Circular Dichroism Measurements

Circular Dichroism (CD) was measured on a JASCO CD J1000 spectrometer. Samples were diluted in 20 mM Tris.HCl, 20 mM NaCl pH 7.4, to a final concentration of 4 μM. Measurements were performed at 25°C using wavelengths ranging from 185 to 260 nm in a span of 100 mdeg at a scanning speed of 20 nm/min. Measurements from 10 experiments were accumulated. CD plots were drawn based on the observed values of CD measurements and concentrations used in measurements.

### IC_50_ Assay of Linear and Cyclic Peptides

TheDUB-mediated hydrolysis of fluorogenic Ub-rhodamine was measured over time in a fluorescence intensity assay using a buffer containing 25 mM Tris.HCl, 100 mM NaCl, 5 mM DTT, 0.05% Tween-20 and 0.1 mg/ml BGG at pH 8.0 and at 37°C (Lavis et al., 2006; Sahtoe et al., 2015). In the case of the IC_50_ assay for Rpn11, we set up a Fluorescent Polarization (FP) assay using 2 µM Ub-FP substrate in an assay buffer containing 50 mM HEPES.NaOH, 100 mM NaCl, 100 mM KCl, 1 mg/ml CHAPS and 0.5 mg/ml BGG at pH 8.0 (Hameed et al., 2019). Fluorescence intensity was monitored at an excitation wavelength of 487 nm and an emission wavelength of 535 nm using a BMG PHERAstar^®^ FSX plate reader. For the FP assay, the FP was monitored at 0° and 90° relative to the polarization of the incident beam at an excitation wavelength of 540 nm and an emission wavelength of 590 nm using a BMG PHERAstar^®^ FSX plate reader. The enzymes UCH-L1, UCH-L3, and BAP1 were expressed and purified in the lab using established protocols. UCH-L5 was obtained commercially from Novus Biologicals (CAT#: NBP1-72315). The parent enzyme stock of UCH-L5 was diluted into the assay buffer at a final concentration of 1 nM. For UCH-L1, UCH-L3 and BAP1, the final enzyme concentration was 100 pM, 20 pM, and 1 nM, respectively. Rpn11 was expressed in-house as a heterodimeric Rpn11/Rpn8 complex and used at a final concentration of 1 µM (Hameed et al., 2019). The Ub-Rhodamine110-MP substrate was prepared synthetically according to the previously reported procedure (Kooij et al., 2020), dissolved in DMSO, and further diluted in Milli-Q (MQ) water and subsequently in assay buffer to a final concentration of 100 nM. The Ub-FP substrate was also prepared according to established protocols (Geurink et al., 2012). The Ub_1-17_ peptides were dissolved in DMSO as a 5 mM stock solution and stored at −20°C. The DMSO stock was diluted in MQ water and further diluted into assay buffer before use. Serial dilutions of the peptides were prepared in assay buffer, ranging from 100 to 0.1 µM. The inhibitor/enzyme mixture was first prepared in 384-well Corning™ low volume flat-bottom plates and incubated at 37°C for 60 min. Following the incubation of the enzyme with inhibitor, the substrate was then added to the mixture and immediately measured in the plate-reader for 90 min at 37**°**C.

### Activity-Based Competition Assay

Recombinant UCH-L1, UCH-L3, UCH-L5, and BAP1 were diluted at a concentration of 1 μM in a labeling buffer containing 25 mM Tris pH 7.5, 100 mM NaCl, and 5 mM DTT. For the UCH-L5 competition assay, cyclic peptide **2** was added at different concentrations ranging from 0.2 to 100 µM and incubated for 60 min at 37**°**C. Subsequently, 0.5 µM of fluorescent Cy5-UbVME probe was added and incubated for 2 min at 37**°**C. For UCH-L1, UCH-L3, UCH-L5, and BAP1 competition assay, **2** was added at 10 µM concentration for 60 min at 37**°**C followed by incubating with 0.5 µM of Cy5-UbVME or 0.5 µM of Rho-UbPA for 5 min at 37**°**C. The reaction was stopped by the addition of reducing sample loading buffer (Invitrogen, Cat# N0007) and boiled before running on 4–12% SDS-PAGE agarose gel using MOPS buffer. A fluorescence scan was made of the gel on a Typhoon FLA 9500 (GE Healthcare Lifesciences) using filters set for Cy5 (λ_ex_/λ_em_ 640/680 nm) or Rhodamine (λ_ex_/λ_em_ 480/520 nm). Coomassie staining was carried out using InstantBlue™ Protein Stain (Sigma-Aldrich).

### Activity-Based Deubiquitinating Proteases Profiling in Cell Lysate

MCF7 cells were seeded into a 10 cm dish. The next day cells were transfected with HA-UCH-L5 plasmid (pcDNA3-HA-Uch37 was a gift from Joan Conaway & Ronald Conaway (Addgene plasmid # 19415; http://www.addgene.org/19415/; RRID: Addgene_19415 (Yao et al., 2006)) by using PEI transfection reagent (Polysciences, Cat# 23966–1). MCF-7 cells expressing HA-UCHL5 were first lysed with lysis buffer containing 50 mM Tris HCl pH 8.0, 150 mM NaCl, 5 mM MgCl_2_, 5 mM DTT, 0.8% NP40, and a cocktail of protease inhibitors (cOmplete EDTA-Free Protease Inhibitor Cocktail (54925800; Roche)) by incubating them in a cold room for 30 min. Lysed cells were centrifuged at 20,000 rcf for 20 min at 4**°**C. Cyclic peptide **2** was added to cell lysate at different concentrations ranging from 0.2 to 100 µM and incubated for 60 min at 37**°**C. Subsequently, 0.5 µM of fluorescent Cy5-UbVME probe was added and incubated for 5 min at 37**°**C. The reaction was stopped by the addition of reducing sample loading buffer (Invitrogen, Cat# N0007) and boiled before running on 4–12% SDS-PAGE agarose gel using MOPS buffer. A fluorescence scan was made of the gel on a Typhoon FLA 9500 (GE Healthcare Lifesciences) using filters set at 600 nm (excitation wavelength) and 640 nm (emission wavelength). Following the fluorescent scan, proteins transfer to the nitrocellulose membrane. Endogenous UCH-L5, HA-UCH-L5, and actin (as a loading control) protein levels were analyzed by western blotting using rabbit anti-UCH-L5 (Abcam, Cat# ab124931, in 1:5000 dilution), mouse anti-HA (BioLegend, 901514, in 1:1,000 dilution) and mouse anti-β-actin (Sigma-Aldrich, Cat# A5441, in 1:10000 dilution) antibodies as primary antibodies. IRDye 680LT goat anti-rabbit IgG (H + L) (Li-COR, Cat# 926–68021, 1:20,000) and 800CW goat anti-mouse IgG (H + L) (Li-COR, Cat# 926–32210, 1:5000) were used as secondary antibodies. The signal was detected using direct imaging by Odyssey^®^ CLx Infrared Imaging System (LI-COR).

### Western Blot Analysis of Ubiquitinated Substrates

MCF7 cells were seeded in 6-well plates 24 h before incubation with peptide **2** at 5 μM, 10 μM, and 25 µM final concentrations for 24 h, 1 µM final concentration of bAP15 (UCH-L5 and USP14 inhibitor) for 3 h (Fukui et al., 2019) or 5 µM MG132 (26S Proteasome inhibitor) for 4 h (Gavilan et al., 2015). DMSO was used as vehicle control. Following the end of the incubation times, MCF7 cells were harvested using 3X NuPAGE SDS Sample buffer (Invitrogen, Cat# NP0007) containing β-mercaptoethanol and 1x DNAse and sonicated in Bioruptor Pico sonicator (Diagenode) (10-sec pulse on, 10 s pulse off for 10 cycles). After boiling them at 95**°**C for 10 min, the samples run on 4–12% SDS-PAGE agarose gel using MOPS buffer. Proteins were transferred to the nitrocellulose membrane. Ubiquitinated substrates were detected using a mouse anti-ubiquitin (P4D1) antibody (Santa Cruz, Cat# sc-8017). Mouse anti-β-actin (Sigma-Aldrich, Cat# A5441, in 1:10000 dilution) was used to detect actin as a loading control. 800CW goat anti-mouse IgG (H + L) (Li-COR, Cat# 926–32210, 1:5000) were used as secondary antibodies. The signal was detected using direct imaging by Odyssey^®^ CLx Infrared Imaging System (LI-COR). The experiment was repeated in triplicates. The total amount of ubiquitinated substrates and the actin level was quantified using Licor Image studio densitometry software, and the total level of ubiquitinated substrates was normalized to the actin level.

### Cell Viability Assay

MCF7 cells were seeded at a density of 5,000 cells/well on a 96-well plate 24 h prior to incubation with peptide **2** at the indicated final concentrations for 24 and 48 h. DMSO was used as vehicle control. Cell viability was determined by using the CellTiter-Blue^®^ Reagent assay (Promega, PR-G8080). CellTiter-Blue^®^ Reagent is added directly to each well in 1:20 dilution, MCF7 cells were incubated at 37°C to allow cells to convert resazurin to resorufin. The fluorescent signal was measured at λ_ex_/λ_em_ 560nm/590 nm.

### Statistical Analysis

Statistical evaluations report on Student’s *t*-test (two-tailed distribution). * indicates significant differences as followed *p < 0.05, **p < 0.01, and ***p < 0.001 while ns shows not significant). All error bars correspond to the mean ± SD.

## Results

### Design, Synthesis and Validation of Ub_1-17_ Beta-Sheet Peptides

**FIGURE 1:**
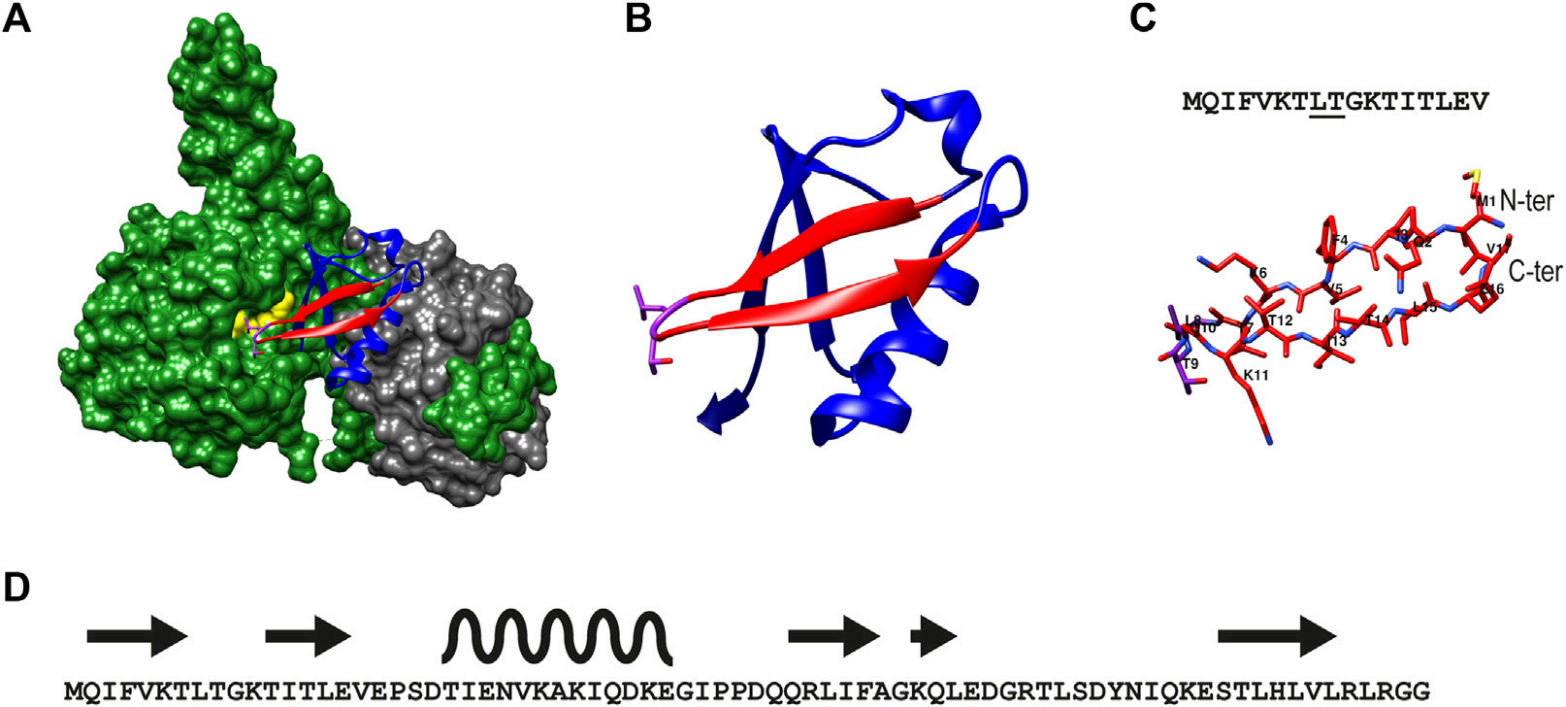
Illustration of the structure of β-sheet hairpin of Ub used in this study **(A)** The structure of UCH-L5 (in green) in complex with Ubiquitin (in blue) and Rpn13 (in grey) as reported (PDB: 4UEL). Residues 1–17 of Ub are colored in red and the residues Leu8, and Thr9 of Ub are colored in pink, and Leu220 and Glu34 of UCH-L5 are colored in yellow. **(B)** Ribbon structure of Ub showing the β-sheet hairpin enclosing residues 1 to 17 and the interacting residues Leu8 and Thr9 in pink. **(C)** The secondary structure of β-sheet hairpin consisting of residues 1 to 17 is used in this study as an inhibitor for UCH-L5.The residues Leu8 and Thr9 are underlined in the sequence (top panel). **(D)**. The overall sequence of Ub (1–76) shows the positions of β -sheet hairpins (in arrows) and α-helix (in wave).

The X-ray structure of UCH-L5 in complex with regulatory proteins Rpn13 and INO80G has been elucidated (Sahtoe et al., 2015), revealing the binding mode of Ub to UCH-L5 (Figure 1A). UCH-L5 by itself has significant deubiquitination activity *in-vitro* when tested in Ub-based fluorescence assays. In cells, Rpn13 was shown to augment the activity of UCH-L5 by providing additional recognition elements of Ub thereby reorienting the C-terminus of Ub into the active site of UCH-L5. On the other hand, INO80G completely abolishes the deubiquitinating activity of UCH-L5 by mimicking Ub and blocking the Ub binding site (Sahtoe et al., 2015). It can be appreciated from the crystal structures of UCH-L5:RPN13:Ub (PDB:4UEL), that Ub binds to UCH-L5 using its hydrophobic patch consisting of residues Leu8, Thr9, and Ile44 in addition to its C-terminal tail (Figures 1A,B). The N-terminal β-hairpin of Ub, comprising residues 1 to 17, can be seen in direct contact with the hydrophobic Leu38 pocket of UCH-L5. In addition to Leu8 and Thr9, residues Phe5 and Ile13 were implicated to be essential in the binding of Ub to UCH-L5. This information provided the rationale for designing a peptide-based inhibitor that includes Leu8 and Thr9 (Figure 1C). We first tested the length of the peptide that can inhibit UCH-L5 using a Ub-rhodamine substrate using a plate reader assay. In this assay, the DUB cleaves the amide bond of the fluorogenic substrate connecting rhodamine to the C-terminal G76 of Ub and a fluorescent signal is evoked. Hence monitoring the increase in fluorescence over time can be directly correlated to the activity of the DUB. If an inhibitor can slow down or completely inhibit the DUB activity, this will result in a slower increase or even complete absence of increasing fluorescence. According to the data obtained, only the β-hairpin peptide encompassing residues 1 to 17 inhibited UCH-L5 at 50 µM concentration while other truncated peptides did not (Supplementary Figure S1). Although full-length Ub has robust structural stability, it is unknown if the β sheet peptide Ub_1-17_, by itself in its linear form, would retain its structural integrity in solution. Therefore, in addition to the end-protected linear β-sheet peptide (**1**), we designed a cyclized β-sheet peptide (**2**) that is known for its resistance to intrinsic exopeptidase activity in cells (Joo, 2012). We also designed a control cyclic peptide, **3**, lacking the crucial Leu8, Thr9 interaction by replacing it with Gly8 and Gly9.

**FIGURE 2:**
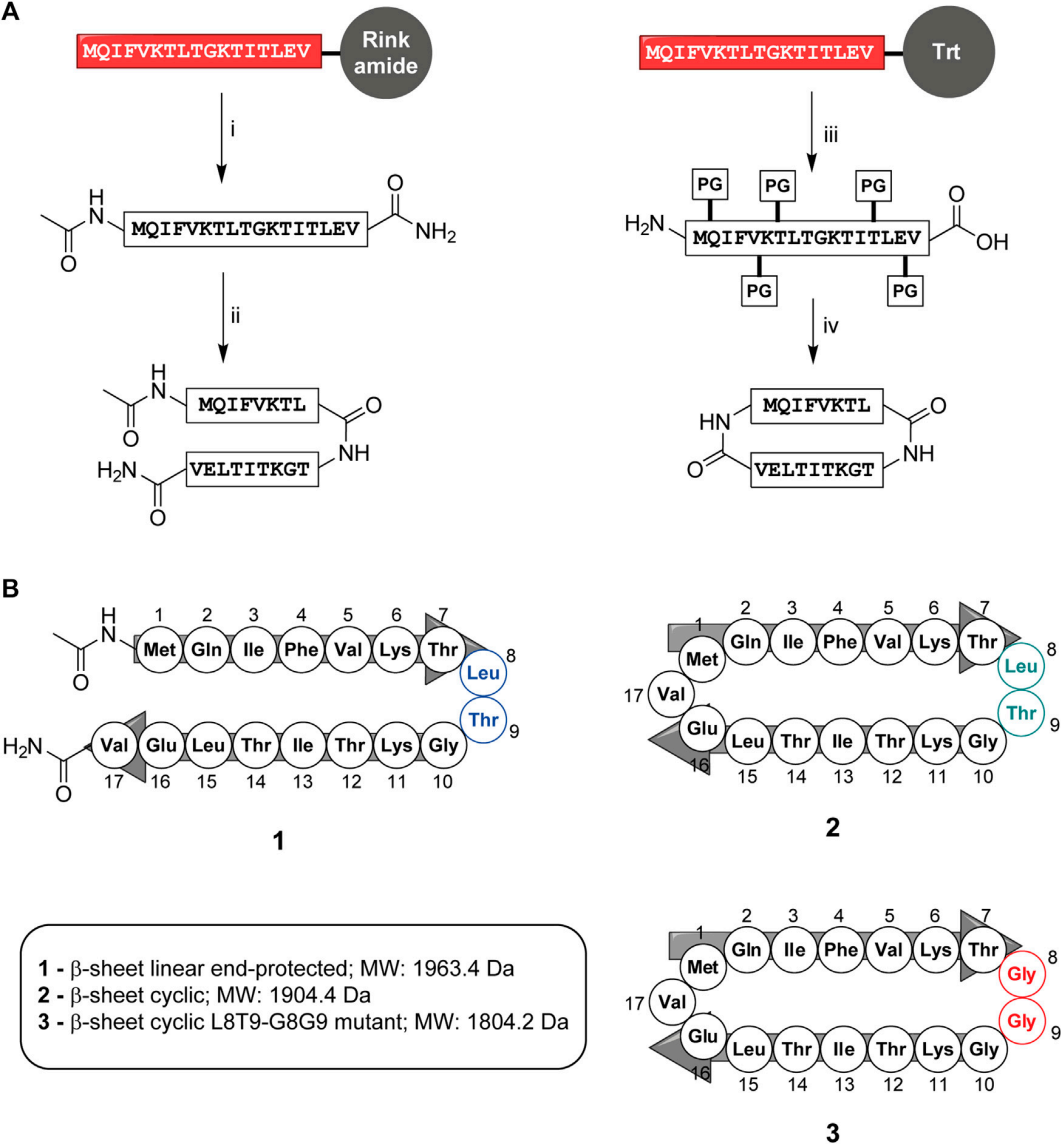
**(A)** Synthesis of end-protected linear peptide, **1**, and cyclic peptide, **2**: i) Acetic anhydride followed by removal of the protective group and release from the resin using TFA/TIS/Phenol/H_2_O ii) RP-HPLC, lyophilization and refolding from DMSO to buffer iii) HFIP/DCM (1:4 v/v), iv) PyBOP, DIPEA in DMF followed by total deprotection using TFA/TIS/Phenol/H_2_O, RP-HPLC, lyophilization and refolding from DMSO to buffer. **(B)** Composition of peptides **1**, **2**, and **3**. Box: Molecular weights of peptides **1**, **2**, and **3**. Both linear and cyclic peptides inhibited UCH-L5.

**FIGURE 3:**
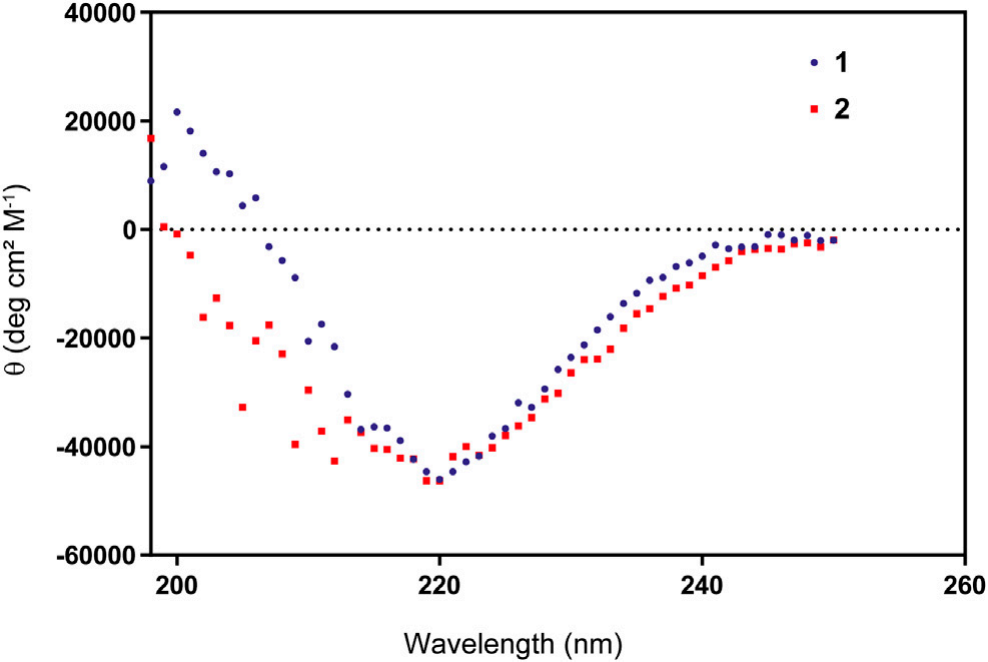
Circular Dichroism of peptides **1** and **2** showing a proper β-sheet hairpin folding pattern.

The linear peptide (**1**) was prepared uneventfully with a yield of 24% after HPLC-purification carrying an acetyl group on the N-terminus and a carboxamide on the C-terminus. The cyclic version of the peptide (**2**) and the control peptide (**3**) were generated by coupling the N-terminus of Met1 to the C-terminus of Val17 from the fully-protected synthetic peptide using PyBOP and DIPEA in DMF at a lower concentration to avoid intermolecular couplings (Figures 2A,B; Supplementary Figure S2). The cyclization reaction was efficient with more than 95% conversion and up to 30% overall yield after HPLC purification. To check for correct folding (Migliore et al., 2020), both **1** and **2** were analyzed using circular dichroism, which revealed a β-sheet conformation in both cases (Figure 3).

**FIGURE 4:**
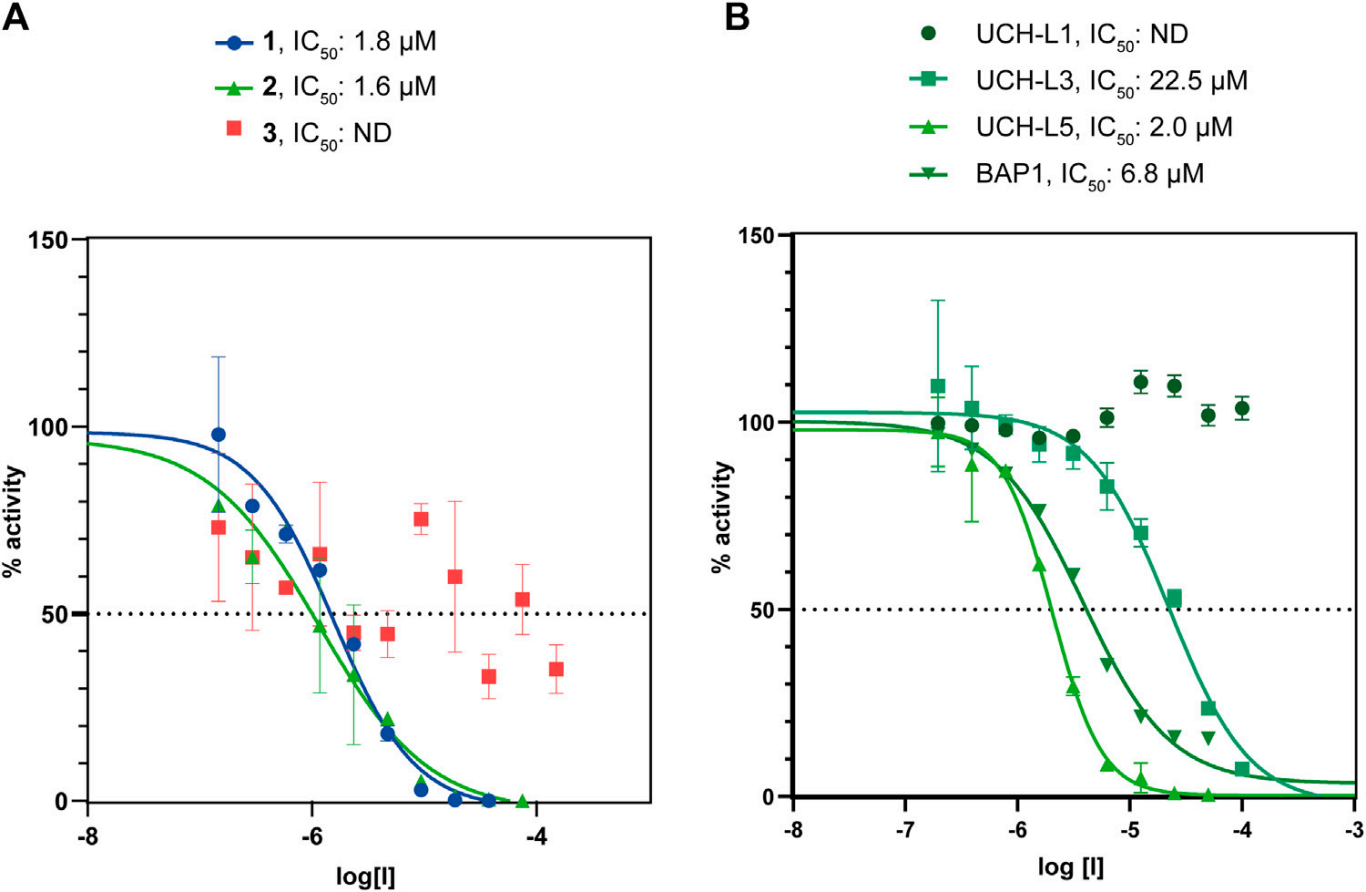
**(A)** Comparison of IC_50_ values of β-sheet hairpin peptides **1**, **2**, and **3**. Peptides **1** and **2** show inhibition in the lower micromolar range. **(B)** Comparison of IC_50_ values of **2** with DUBs belonging to the UCH family, namely UCH-L1, UCH-L3, UCH-L5, and BAP1.

First, we tested whether our peptides **1** and **2** were able to inhibit UCH-L5 activity using a standard Ub-rhodamine substrate assay in a plate-reader. Both **1** and **2** were able to inhibit the activity of UCH-L5 in a concentration-dependent manner while the control cyclic peptide **3** where the crucial interacting amino acids Leu8 and Thr9 were replaced with Gly8 and Gly9 did not inhibit UCH-L5 at all (Figure 4A). The linear peptide, **1**, inhibited UCH-L5 with an IC_50_ of 1.8 µM while the cyclic version, **2**, inhibited UCH-L5 slightly better with an IC_50_ of 1.6 µM (Figure 4A). Since both **1** and **2** were properly folded and inhibited UCH-L5 with comparable IC_50_ values, we confirmed that the cyclic peptide **2** has similar inhibitory potential as the linear version. We proceeded with the cyclic peptide **2** for further validation as cyclization provides an additional layer of structural and proteolytic stability in a cellular environment (Joo, 2012).

### The Selectivity of Cyclic Peptide 2 Towards UCH-L5

UCH family consists of four DUBs, namely UCH-L1, UCH-L3, UCH-L5, and BAP1, which share a common UCH domain and similar binding pocket for Ub (Komander et al., 2009). To check whether the cyclic peptide **2** can interfere with the activity of other DUBs in the UCH family, we measured the IC_50_ of the cyclic peptide **2** for UCH-L1, UCH-L3, and BAP1 along with UCH-L5 in the Ub-Rho assay (Figure 4B). The cyclic peptide inhibits UCH-L3 ten times less efficiently (IC_50_ = 22.5 µM), while BAP1 is inhibited three times less efficiently than UCH-L5 (IC_50_ = 6.8 µM). UCH-L1 activity is completely unaffected even at the highest concentration of 100 µM of the cyclic peptide. Hence we concluded that among the other members of the UCH family, the cyclic β-sheet peptide **2** inhibits UCH-L5 most efficiently.

To further investigate whether the cyclic peptide **2** inhibits the activity of Rpn11, the metalloprotease DUBs found in the 19S cap of the 26S proteasome, we carried out a standard fluorescence polarization assay using a Ubiquitin-Fluorescence Polarization (Ub-FP) substrate containing Ub linked by an isopeptide bond to a TAMRA-labelled Ub peptide, comprising residues 41 to 54 of Ub (Supplementary Figure S3A). We observed that the cyclic peptide, **2**, was unable to inhibit Rpn11/Rpn8 activity (Supplementary Figure S3B), potentially suggesting that the Rpn11 activity remains unhindered in cells in the presence of **2**.

**FIGURE 5:**
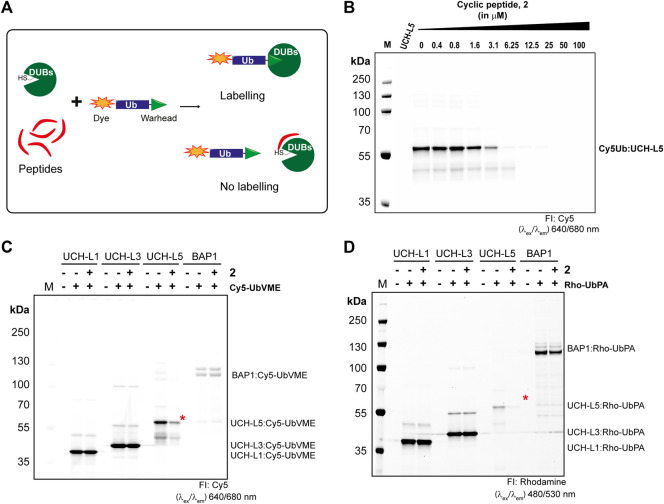
Selectivity of peptide 2 towards UCH-L5 among UCH family members. **(A)** Principle of activity-based competition assay. **(B)** Fluorescence scan showing the labeling of UCH-L5 with Cy5-UbVME in the presence of different concentrations of **2**. **(C,D)** Fluorescence scan showing the labeling of UCH-L1, UCH-L3, UCH-L5, and BAP1 with Cy5-UbVME (in C) or Rho-UbPA (in D) activity-based probes. Cy5-UbVME or Rho-UbPA probe was used to label different enzymes pre-incubated with 10 µM of **2**. The Ub-labeled recombinant UCH-L5 was indicated by red asterisks.

Next, we tested whether our cyclic peptide **2** can compete with the activity-based probe Cy5-UbVME in the labeling of UCH-L5. Cy5-UbVME reacts covalently with the active site cysteine of UCH-L5 using the vinyl group at the Ubs C-terminus (Kramer et al., 2012). Peptide, **2**, in principle, binds at the allosteric site which disrupts binding, and thus the covalent labeling of the enzyme with the Cy5-UbVME probe (Figure 5A). We incubated UCH-L5 with different concentrations of peptide **2** for 60 min at 37°C. Since our inhibitor **2** is non-covalent, we added the covalent Cy5-UbVME probe to the peptide-incubated UCH-L5 only for 2 min at 37°C allowing competition between non-covalent binding **2** and covalent binding Cy5-UbVME. The samples were subsequently quenched with 3X Laemli buffer and run on SDS-PAGE gel and labeling of UCH-L5 was visualized using a fluorescence scanner. The gel shows that the peptide was able to significantly outcompete Cy5-UbVME labeling of UCH-L5 at a concentration higher than 3 µM. (Figure 5B). This modification was also evident from the Coomassie stain of the same gel (Supplementary Figure S4).

Furthermore, we also tested the competitive labeling of other DUBs in the UCH family, namely UCH-L1, UCH-L3, and BAP1. Our results indicate a stronger reactivity of Cy5-UbVME to UCH-L5, L1, and L3 than BAP1, which was successfully labeled with Rho-UbPA (Ekkebus et al., 2013; Mons et al., 2021). Therefore, we used both Cy5-UbVME and Rho-UbPA probes for all the enzymes in our competition assay. Based on the IC_50_ values from Figure 4B, we incubated all enzymes of the UCH family with 10 µM of **2** for 60 min at 37°C followed by incubating with Cy5-UbVME or Rho-UbPA for 5 min at 37°C, and the reaction was stopped by adding sample loading dye and further boiling at 95°C for 10 min. The samples were run on an SDS-PAGE gel and analyzed on the fluorescence scanner. We observed a clear inhibition of UCH-L5 by **2** at 10 µM while the other enzymes showed no inhibition (Figures 5C,D). There was a slight reduction in BAP1 labeling with **2** when using Rho-UbPA (Figure 5D). The finding is consistent with the fact that BAP1 was also inhibited by **2** albeit to a lesser extent than UCH-L5. A similar pattern was also observed in the Coomassie stain of the same gels (Supplementary Figures S5A, S5B). Taken together with the IC_50_ assays, we showed that the peptide inhibitor **2** preferred UCH-L5 at lower micromolar concentrations, even though all UCH enzymes share a similar binding motif to Ub.

### Activity-Based Deubiquitinating Proteases Profiling in Cell Lysate Showed the Selectivity of 2 Towards UCH-L5

**FIGURE 6:**
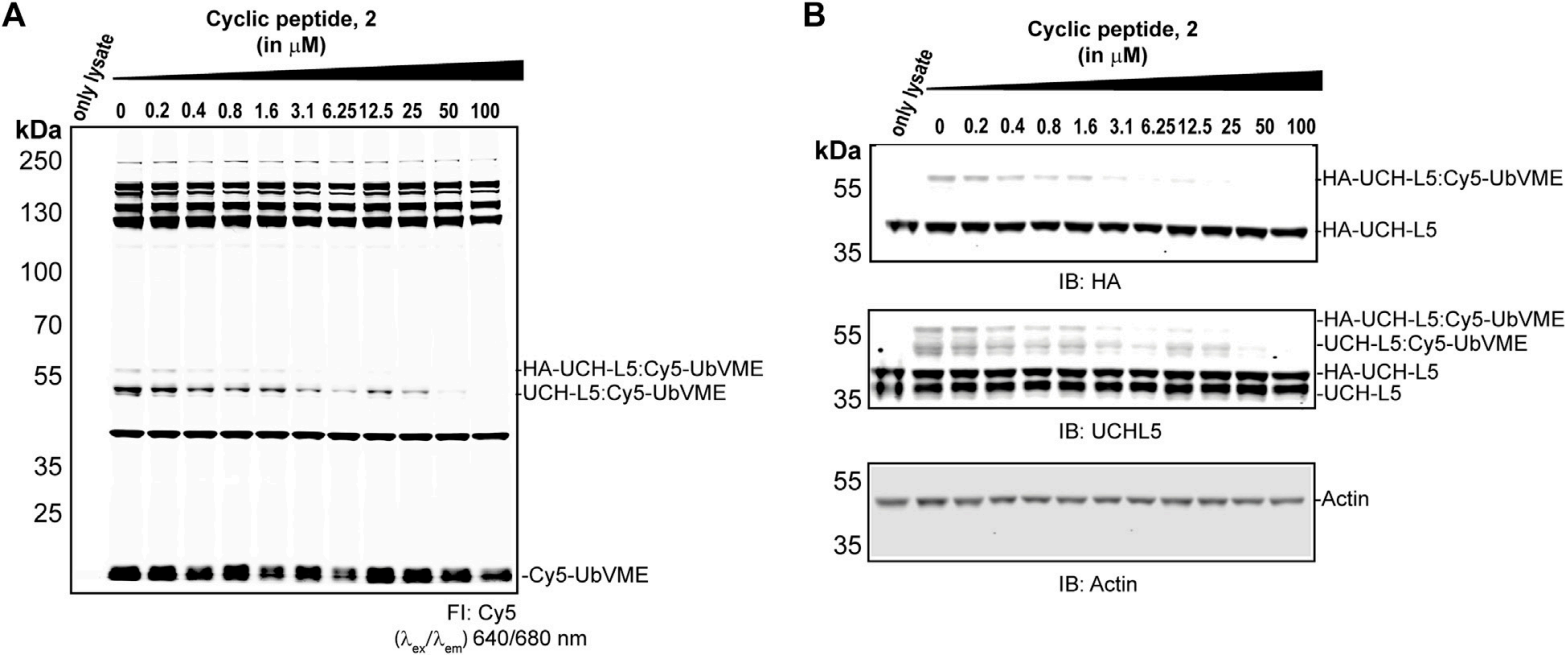
Activity-based DUB profiling in cell lysate in competition with **2**. **(A)** Fluorescence scan showing the labeling of DUBs with Cy5-UbVME in MCF7 cells expression HA-tagged UCH-L5. Lysates from MCF7 cells were added with **2** at indicated concentrations for 1 h, followed by labeling with Cy5-UbVME for 5 min. **(B)** Immunoblots of the same gel using anti-UCH-L5, anti-HA, and anti-β-Actin antibodies.

To investigate the selectivity of **2** among the other DUBs in the cell, we expressed HA-tagged UCH-L5 in MCF7 cells, where endogenous UCH-L5 is also present. The lysates from these MCF7 cells were treated with **2** at the indicated concentrations (0–100 µM) for an hour at 37°C, followed by 5 min incubation with a Cy5-UbVME probe. The bands observed in the fluorescent scan represent the active DUBs in the cell lysate, and the disappearance of the fluorescent band can indicate the inhibition of any DUB activity upon treatment with peptide **2**. We observed the disappearance of HA-UCH-L5 and endogenous UCH-L5 in the immunoblot (Figure 6B) after incubating with **2** in a concentration-dependent manner, while the fluorescent scan (Figure 6A) did not show significant disappearance of any other labeled protein bands caused by peptide **2** up to a concentration of 50 µM. This data suggests a potential selectivity of **2** towards UCH-L5 in cell lysate compared to other cysteine DUBs.

### Effect of Cyclic Peptide 2 in MCF7 Cells

**FIGURE 7:**
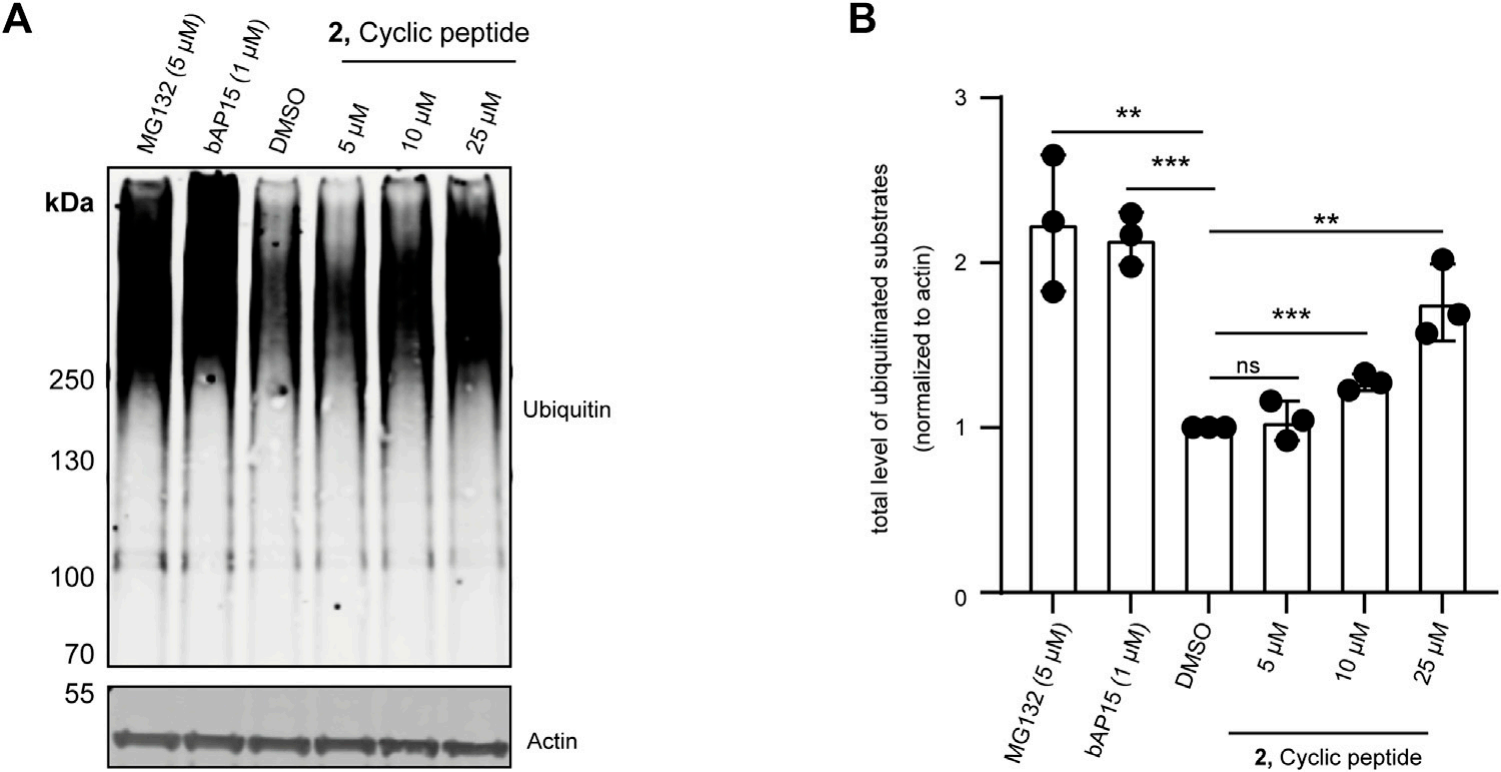
Effect of **2** on overall ubiquitination of MCF7 cells. MCF7 cells were incubated with 5, 10, and 25 µM final concentrations of **2** for 24 h, 1 µM final concentration of UCH-L5, and USP14 specific bAP15 inhibitor for 3 h or 5 µM proteasome inhibitor (MG132) for 4 h. DMSO was used as a loading control. The lysates were run on SDS gel. **(A)** Immunoblot of the gel showing overall ubiquitinated substrates (top panel) and actin levels (bottom panel) using an anti-Ubiquitin and anti-β-actin antibodies, *n* = 3 independent experiments. **(B)** Bar graphs report means of independent measurements of accumulated ubiquitinated substrates (black circles), error bars reflect ±s.d. All significance was calculated using Student’s *t*-test in comparison to the DMSO sample: *p < 0.05, **p < 0.01, and ***p < 0.001.

UCH-L5 is reported to regulate proteasomal degradation of certain proteins by trimming the polyUb chains before the substrate is committed to degradation (Collins and Goldberg, 2017). It has also been shown that inhibiting UCH-L5 would lead to the accumulation of ubiquitinated substrates in cells (Chadchankar et al., 2019). Therefore, to evaluate both the cell permeability of cyclic peptide **2** and its effect on the overall level of ubiquitination in cells, we incubated MCF7 cells with 5 μM, 10 μM, and 25 µM of **2** for 24 h. We also took along the non-selective USP14/UCH-L5 inhibitor, bAP15 (Wang et al., 2014; Fukui et al., 2019), and the 26S proteasome inhibitor, MG132 (Gavilan et al., 2015) as controls. As expected, incubating **2** with MCF7 cells for 24 h increased the level of ubiquitinated substrates, consistent with the result of bAP15 and MG132 treatment (Figures 7A,B) without showing any cell toxicity (Supplementary Figure S6).

Taken together with the previous data from IC_50_ assays, activity-based competition assays, and the activity-based DUB profiling, we showed that the cyclic peptide **2** preferably inhibited UCH-L5 compared to other DUBs in the UCH family and did not show a significant effect on the probe-labeling of other DUBs in MCF7 cell lysate. Furthermore, the cyclic peptide infiltrated cells and consistently resulted in the accumulation of (poly)ubiquitinated substrates similar to that of the previously reported non-specific UCH-L5 inhibitor.

## Discussion

The 26S proteasome is a strategically important complex both in the context of diseases and therapeutic intervention. Many drugs targeting the 26S proteasome have ended up as an effective treatment for diseases such as cancer (Tsvetkov et al., 2018). A well-known treatment for multiple myeloma is Bortezomib which targets one of the proteases residing in the core complex of the 26S proteasome (Frankland-Searby and Bhaumik, 2012). Over the years, several analogs of this compound have been developed as selective inhibitors. However, some of these inhibitors are susceptible to drug resistance over a course of time. Hence alternative therapeutic targets are needed. The evidence so far also indicates the crucial role of UCH-L5 in many diseases (Wicks et al., 2005; Fang and Shen, 2017). The association of UCH-L5 with the 26S proteasome makes it an interesting target for high throughput screening assays. The availability of the structure of UCH-L5 with Ub facilitated the development of a peptide-based inhibitor to target UCH-L5 with better accuracy.

The 26S proteasome is a huge protein complex consisting of many checkpoints to regulate its activity, including the three DUBs associated with it. Although the structure of the 26S proteasomal complex has been elucidated, the exact mode of action of these associated DUBs is a constant subject of debate (Collins and Goldberg, 2017). A few studies suggested that Rpn11, which is located at the base of the lid complex near the core complex, essentially removes the Ub chain en-bloc from the substrate protein as and when the substrate protein is pulled into the core complex (Lander et al., 2012; Worden et al., 2014). Further mechanistic studies are however needed to further prove this mechanism. Moreover, the molecular mechanism and function of the other DUBs are subject to more speculation (Lee et al., 2011; Kim and Goldberg, 2017). It has been proposed that USP14 is essentially a chain-trimming DUB that removes Ub from poly-Ub chains one by one before the substrate is committed to degradation by the proteasome, potentially rescuing those substrates from degradation (Kim and Goldberg, 2017). Only recently has the role of UCH-L5 been put forward as being a K48 specific de-branching DUB, that removes branches from forked Ub chains to facilitate protein turnover (Deol et al., 2020). Not much, however, is known about the specific contribution of UCH-L5 to the proteasome-mediated protein degradation process. With the development of a specific inhibitor for UCH-L5, opportunities to study the functions of all individual DUBs in the 26S proteasome will be possible. Furthermore, the cyclic peptide we developed here can potentially be further developed towards a more potent and selective UCH-L5 inhibitor that might have clinical potential.

## Data Availability

The original contributions presented in the study are included in the article/Supplementary Material, further inquiries can be directed to the corresponding authors.
