# Supplementary material for: Inhibiting UCH-L5: Rational Design of a Cyclic Ubiquitin-Based Peptide Inhibitor
Source: Front Mol Biosci. 2022 May 26;9:866467. doi: 10.3389/fmolb.2022.866467 (PMC9204298; doi:10.3389/fmolb.2022.866467)
Supplement: Supplementary file 1 [file DataSheet1.PDF]

Inhibiting UCHL5: Rational design of a cyclic Ubiquitin-based peptide inhibitor

Dharjath S. Hameed[1], Huib Ovaa[1†], Gerbrand J. van der Heden van Noort[1*] and Aysegul Sapmaz[1*]

Supplementary figures

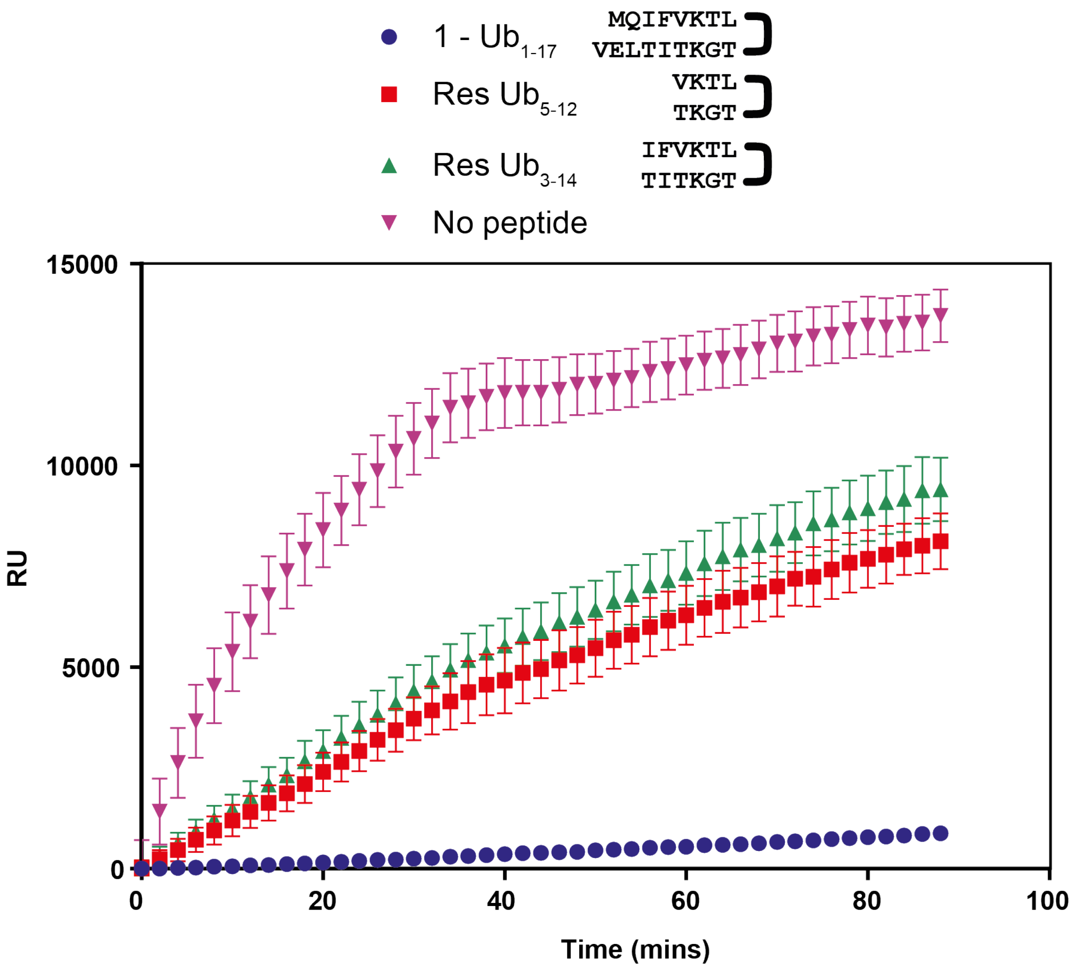

Figure S1: Ub-Rho assay with UCH-L5 against different lengths of Ub β-sheet peptides. Linear end-protected peptides of different lengths were tested for inhibitory property and only the peptide Ub_{1-17} inhibited UCH-L5 at 50 μM concentration.

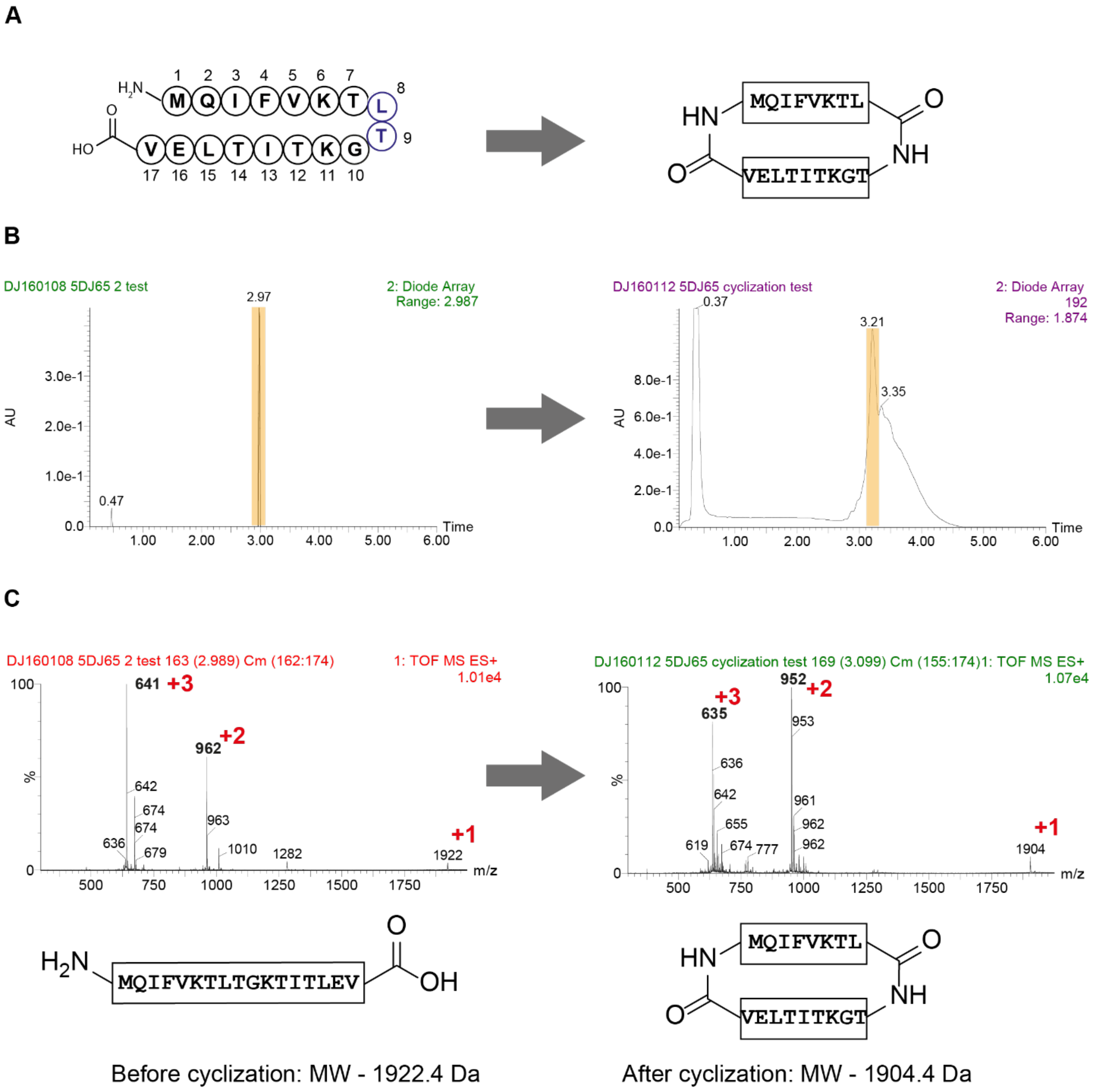

Figure S2 A: Illustration of the cyclization reaction of $Ub_{1\text{-}17}$ peptide. **B:** UV chromatogram of cyclization reaction showing the starting material and product at different retention times. C: Mass spectrum of cyclization reaction showing the loss of water (18 Da) after cyclization reaction is completed. The calculated mass is shown below.

A

B

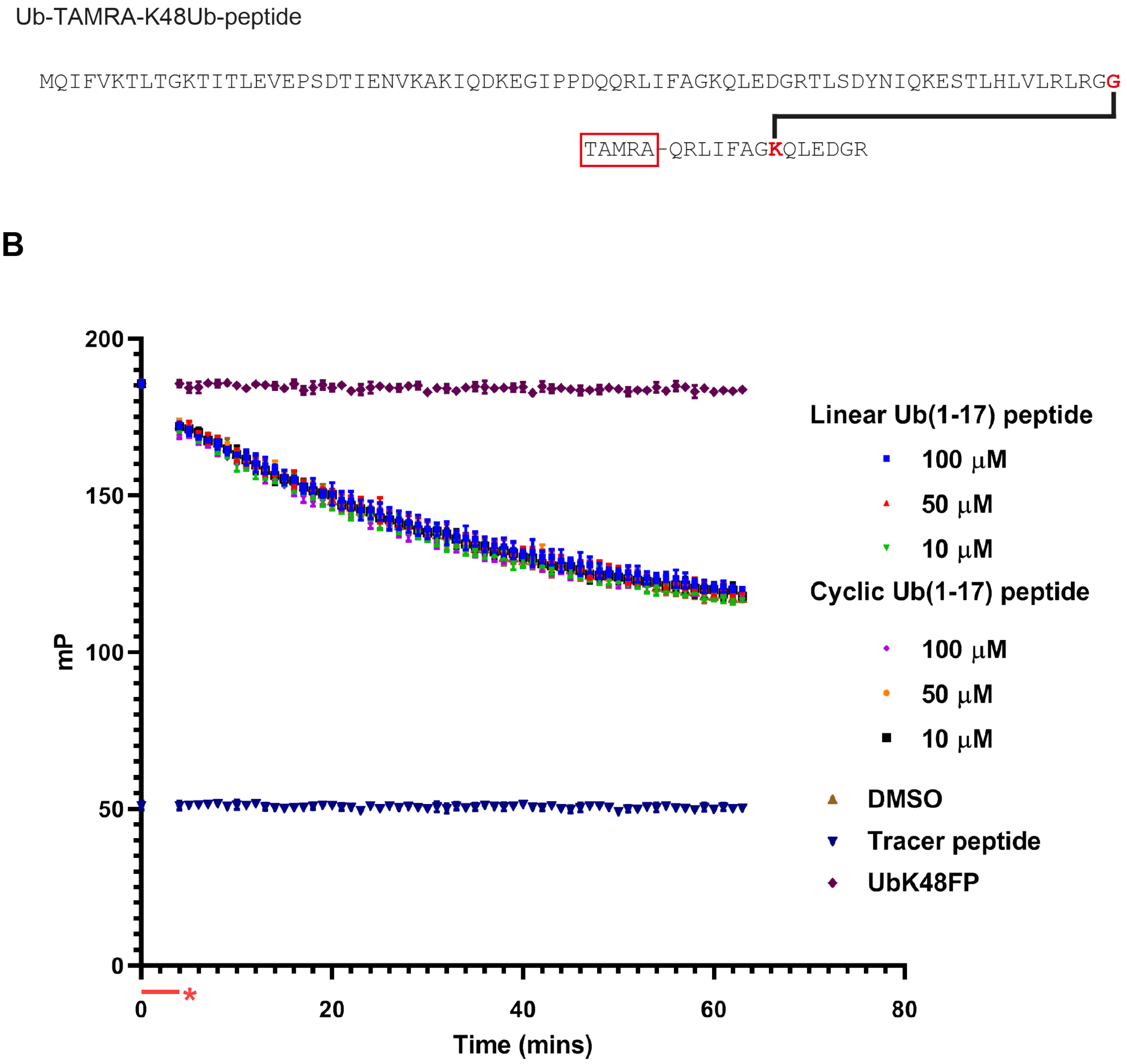

Figure S3: A: Sequence of U-TAMRA-K48Ub-peptide (UbK48FP) substrate used in Rpn11 enzyme activity assay. **B**: Fluorescence polarization data of Rpn11/Rpn8 activity in the presence of different concentrations of both linear and cyclic Ub (1-17) peptides. UbK48FP reagent was used as a substrate and TAMRA-K48(Ub) peptide was used as a tracer peptide. The first four minutes were not measured due to the time taken from adding substrates to measuring the plate.

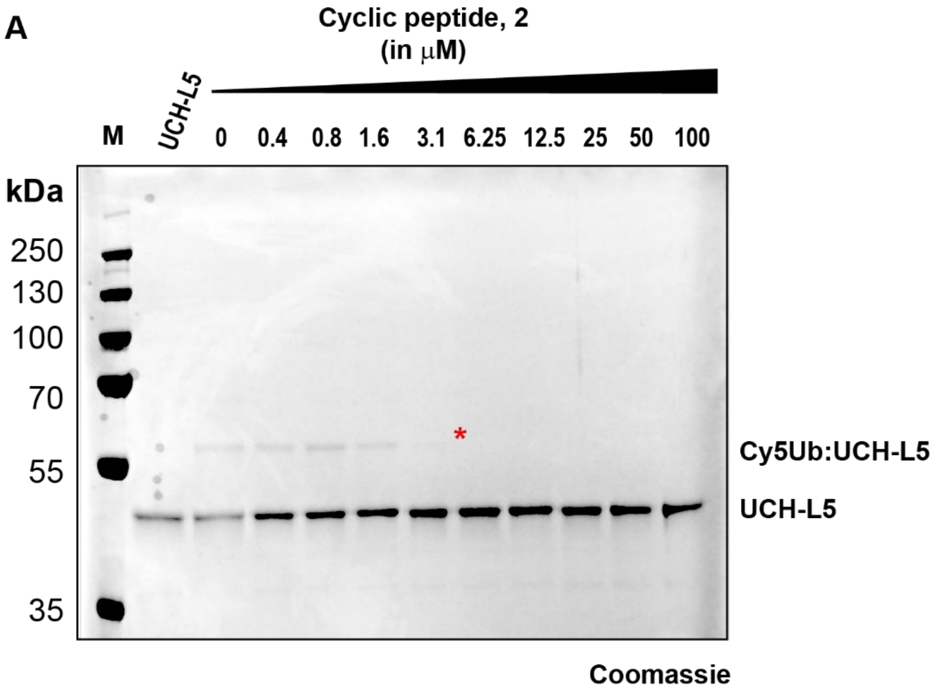

Figure S4: Coomassie stain of gel in figure 5. Asterix shows the presence of labeled UCH-L5 with Cy-UbVME probe.

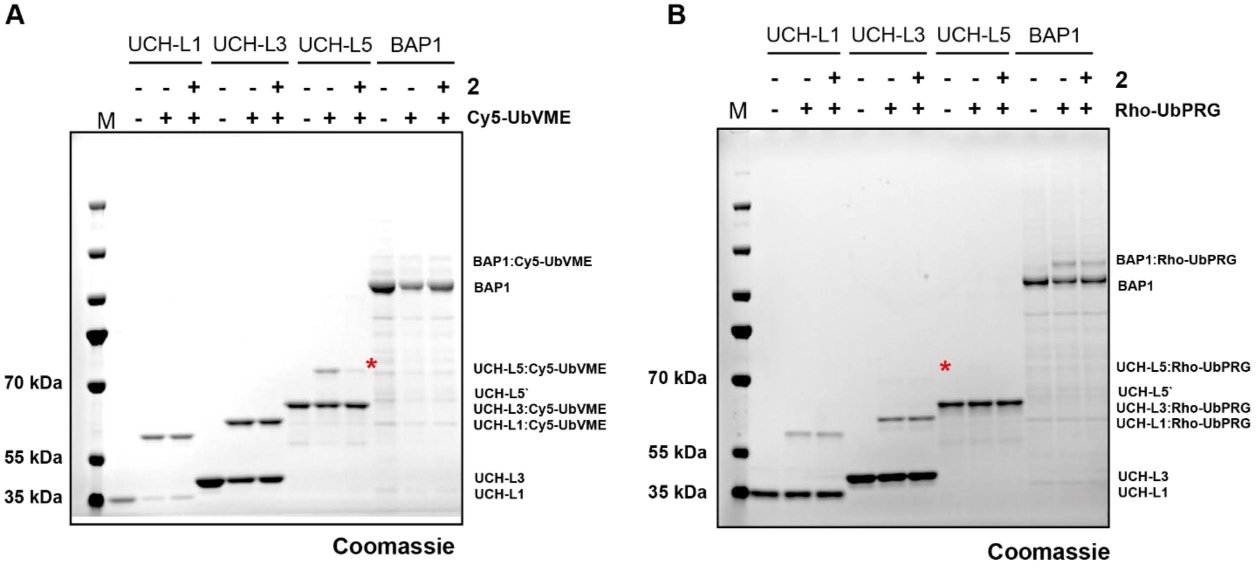

Figure S5: Coomassie stain of gel in figure 6. Asterix marks the position of UCH-L5 labeled with activity-based probes.

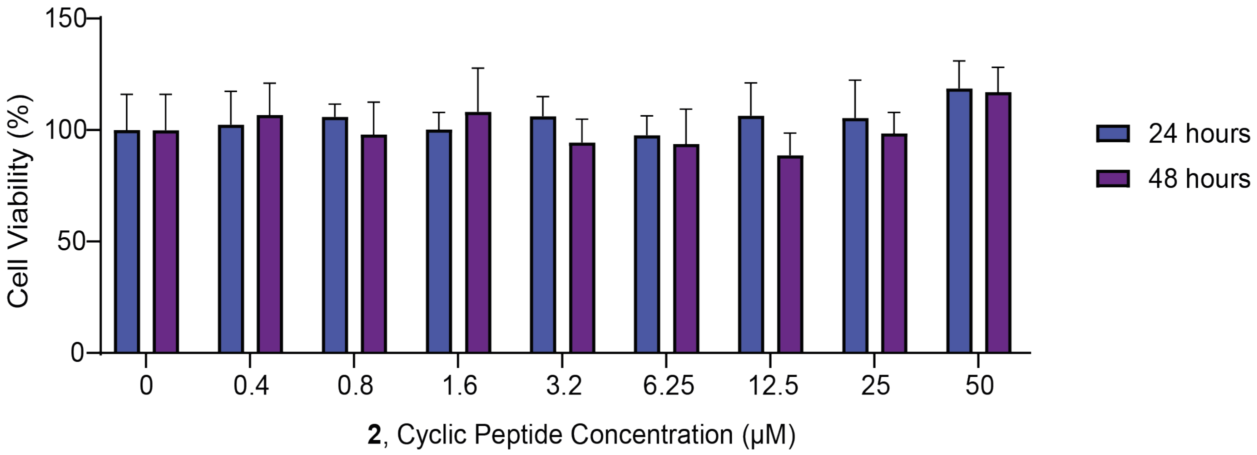

Figure S6: MCF7 Cell toxicity assay with cyclic peptide **2**.

A

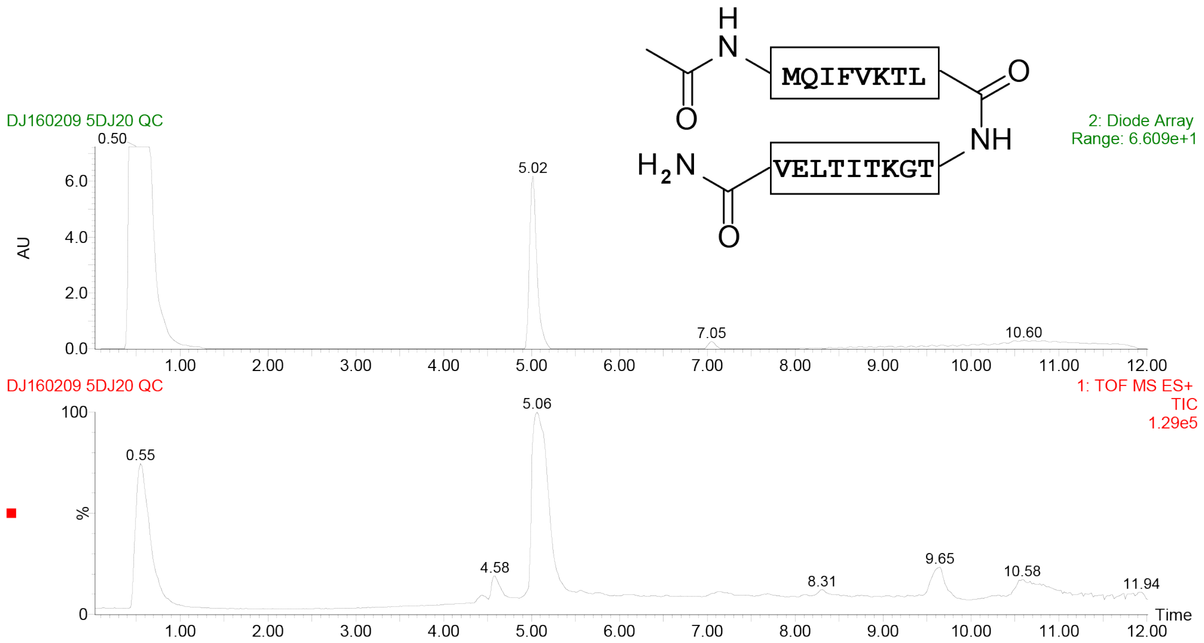

B

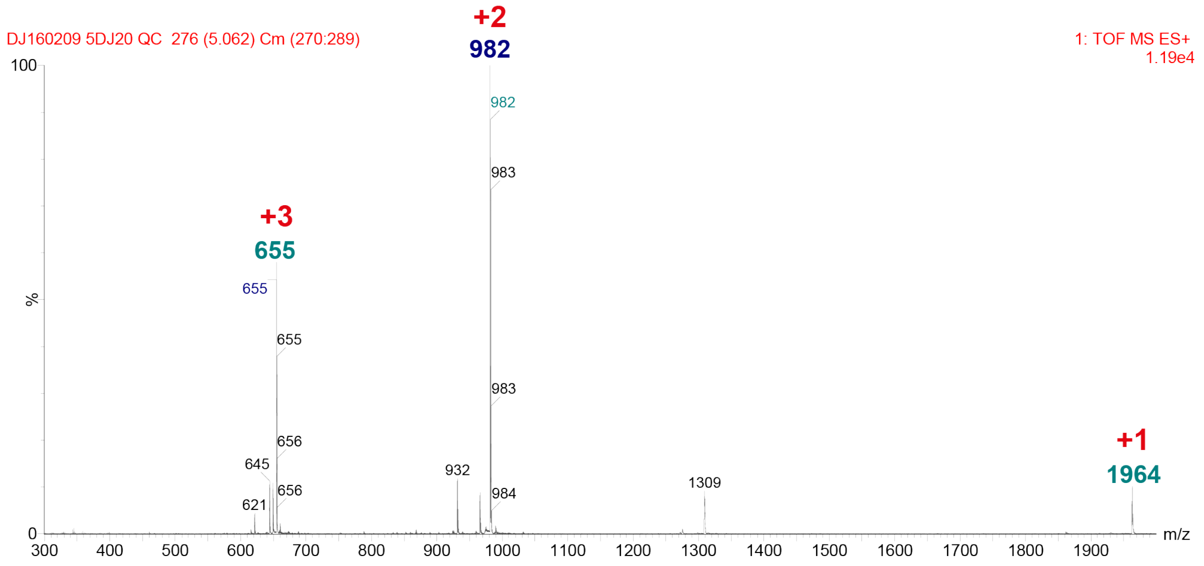

Figure S7: LC-MS analysis of linear Ub (1-17) peptide, **1**. A: Top: UV chromatogram; Bottom: Combined Mass spectrum; Inset: illustration of the linear peptide. B. Combined mass spectrum of UV peak at 5.02 min. Calculated mass: 1963.4 Da. Observed mass: 1964(M+1), 982 (M+2), and 655 (M+3).

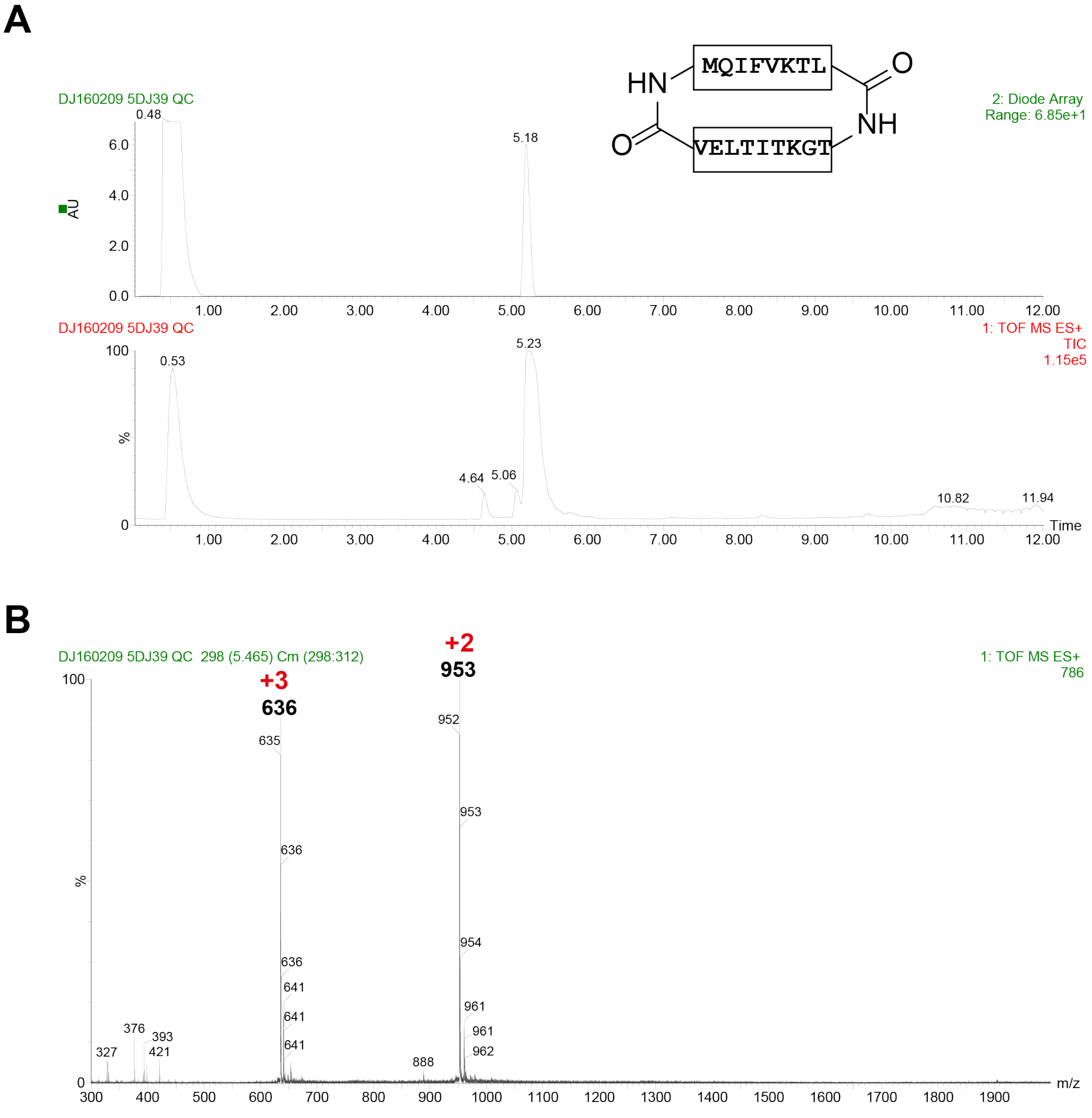

Figure S8: LC-MS analysis of cyclic Ub (1-17) peptide, **2**. A: Top: UV chromatogram; Bottom: Combined Mass spectrum; Inset: illustration of the cyclic peptide. B. Combined mass spectrum of UV peak at 5.18 min. Calculated mass: 1922.4 Da. Observed mass: 953 (M+2) and 636 (M+3).

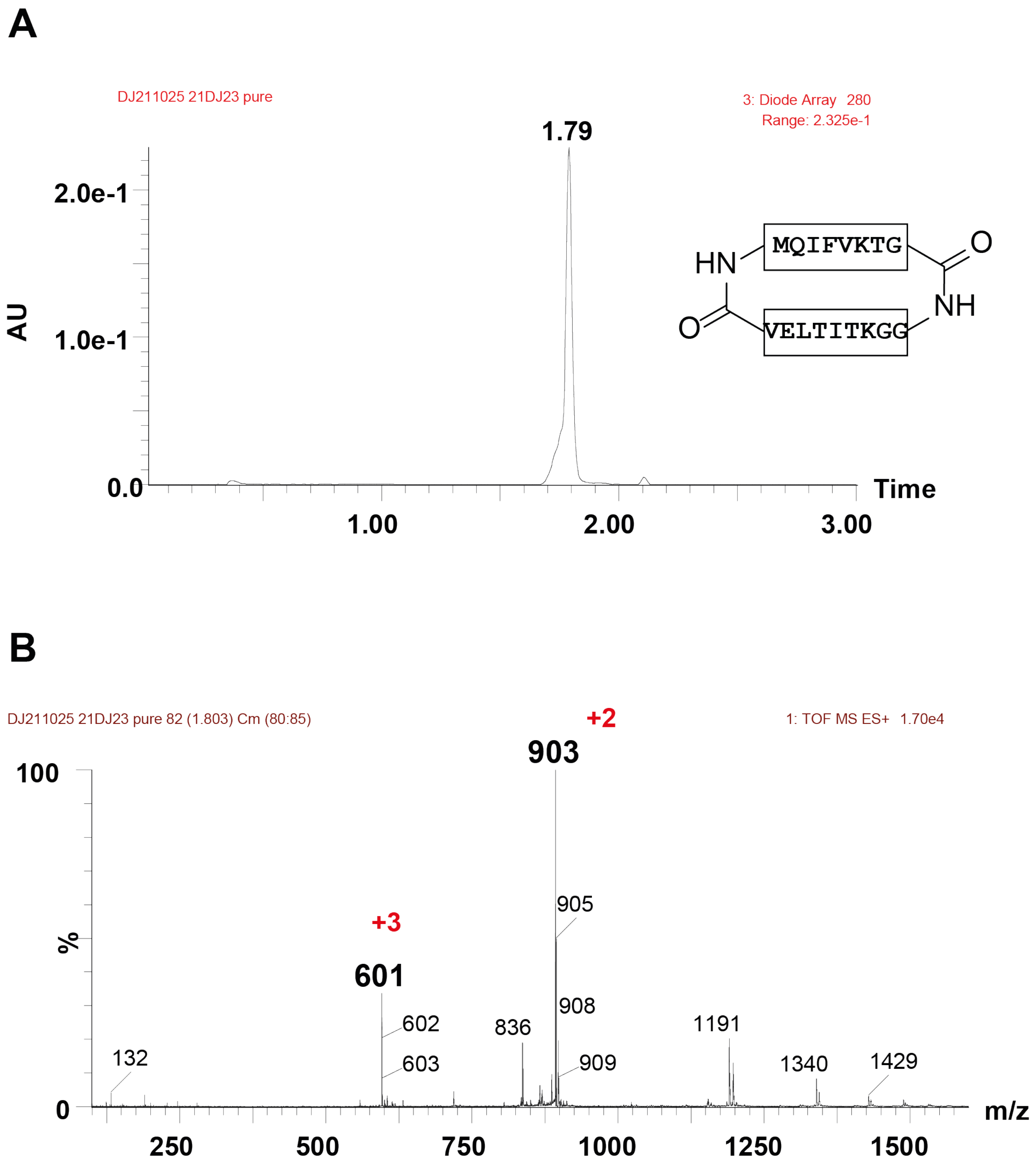

Figure S9 LC-MS analysis of cyclic mutant (LT-GG) Ub (1-17) peptide, **3**. **A:** UV chromatogram; Inset: illustration of the mutant cyclic peptide. B. Combined mass spectrum of UV peak at 1.79 min. Calculated mass: 1804.4 Da. Observed mass: 903 (M+2) and 601 (M+3).
